# PM_2.5_ and ultrafine particles in passenger car cabins in Sweden and northern China—the influence of filter age and pre-ionization

**DOI:** 10.1007/s11356-020-09214-0

**Published:** 2020-05-30

**Authors:** Dixin Wei, Filip Nielsen, Lars Ekberg, Anders Löfvendahl, Maria Bernander, Jan-Olof Dalenbäck

**Affiliations:** 1 Volvo Car Corporation, Gothenburg, Sweden; 2 Division of Building Services Engineering, Department of Architecture and Civil Engineering, Chalmers University of Technology, Gothenburg, Sweden

**Keywords:** PM_2.5_, UFP, Ionization, Filter, Vehicle cabin, Airflow, Recirculation

## Abstract

The main aim of the study was to evaluate the influence of filter status (new and aged), pre-ionization, on the particle filtration in modern passenger cars. Measurements of in-cabin and outside PM_2.5_ (dp < 2.5 μm) concentration and UFP (ultrafine particle, dp < 100 nm) counts, to calculate I/O (indoor to outdoor) ratios, were performed. They were done at two locations, to study the influence of different outside conditions on the HVAC (heating, ventilation, and air-conditioning) system. The measurements were performed in two new cars, with similar HVAC systems and settings, using a new filter and an aged synthetic filter. Furthermore, an ionization unit was installed upstream of the filter in both cars. This enabled the study of filter status, with and without ionization, under common driving conditions. The results show that the HVAC system performances were very similar at the two locations, with average I/O ratios of 0.35–0.40 without ionization and 0.15–0.20 with ionization applied, although the outside conditions were considerably different. Furthermore, the aged filter clearly worsened the filtration ability. Considering the corresponding average PM_2.5_ I/O ratios in one location as an example, the average for the new filter was 0.20 and 0.60 for the aged filter. The corresponding UFP I/O ratios were 0.24 and 0.57. Other findings are that the aged filter with ionization reached a performance close to the new filter (without ionization), and that increased ventilation airflow and decreased recirculation degree, as expected, led to an increase in the I/O ratio for both particle sizes.

## Introduction

During the last 50 years, we have seen an increased population with increased living standards, followed by an increased number of plants for electricity generation and an ever-growing demand for transports, to a large extent based on fossil fuels. This has led to air quality problems, especially increased number of airborne particulate matter.

Epidemiology studies have shown that high particle concentrations influence human health. Gan et al. (2011) stated correlations between exposure to PM_2.5_ (particles of aerodynamic diameter less than 2.5 μm) and risks of respiratory and cardiovascular diseases. UFPs (ultrafine particles, which have aerodynamic diameter less than 100 nm) can more easily deposit in the lung alveoli (Mitsakou et al. 2007), cause greater inflammatory response and move to other organs (Oberdörster 2000). Vehicle passengers are specifically challenged with pollutants from dense surrounding traffic, where elevated particle concentration could exist (Ramos et al. 2016), and also attributable to UFPs from traffic exposure potentially damage lung function (McCreanor et al. 2007).

Nowadays, main protection against outdoor pollutions in vehicles is provided by HVAC (heating, ventilation, and air-conditioning) system, advanced filters, recirculation, incoming air sensors and improved cabin air tightness. Multi-layer filters containing active carbon, electrostatic filter, and two-step filters exist in different cars. However, filter causes a pressure drop which increases as dust loading builds up, and thus deterioration of particle removal. Besides, filters normally provide lower removal at the most penetrating particle size around 100–300 nm. Thus, there is a need to know current air quality in vehicles and the influence of parameters, to support the development in the vehicle industry.

Particle level in-cabin has been studied in private cars, trams and other vehicles. The study scope mainly included PM_2.5_, and sometimes UFP. The focus in the studies was on variation of transportation microenvironments (Kaur et al. 2005; Knibbs and de Dear 2010; Huang et al. 2012; Both et al. 2013; Qiu et al. 2017), influence of ventilation settings (Zhu et al. 2007; Knibbs et al. 2010; Abi-Esber and El-Fadel 2013; Jain 2017), influence of the surrounding traffic (Knibbs et al. 2009), and measurement methods (Kumar et al. 2018). Basic information about these comparative studies is summarized in Table 4 in Appendix. Except for urban and highway measurements, some studies in road tunnels were able to expose vehicles to excessive particle levels and discovered elevated exposure to UFPs in the tunnel compared with urban outdoors (Kaminsky et al. 2009; Knibbs et al. 2010; Nayeb Yazdi et al. 2019; Qiu et al. 2019).

Ambient particle concentration and filter ageing have been recognized to have dominant influence on in-cabin particle concentrations, while aforementioned studies seldom compared different filter statuses and locations.

Pre-ionization of particles has been proven to increase the filtration performance. Several studies presented improvement of filtration efficiency in test rigs or chambers, by 5–70% units (Agranovski et al. 2006; Park et al. 2011; Shi 2012). Ionization-combined filtration already stands for a large market share of air cleaners in building appliances (Kim et al. 2017a), while application in vehicle environments is not common.

Thus, by performing vehicle on-road measurement, this study aimed to expand the understanding of in-cabin and outdoor levels of particles and gain knowledge about the influence of filter status and pre-ionization. The measurements were carried out at two sites—in a road tunnel in Sweden and on-road in Northern China—with different outdoor levels of particles. All measurements were based on the use of the same filters and similar HVAC systems. The measurements were further carried out for a new filter, as well as an aged filter, in both cases without and with pre-ionization. Different ventilation airflow and recirculation degrees were tested as well. Controlled climate settings, such as air conditioning (AC) temperature and air distribution in different vents, were used during all testings.

## Methods

### Measurement area and vehicle

The data utilized in this study were obtained from two measurement campaigns, to achieve extended data ranges in different ambient particle concentration levels. Winter testing in China and summer testing in Sweden also represented Asian and European environments.

Gothenburg city is located on the west coast of Sweden. By year 2018, this second largest city in Sweden reached a population of around 571,900 people; the annual average atmospheric PM_2.5_ concentration was reported to 7.7 μg/m^3^ from the local environment monitoring station (Gothenburg Municipality 2018). In total, 250,483 motor vehicles were in use in year 2018, 76% of the total vehicles were passenger cars, and goods vehicles accounted for 9%; within passenger cars, 56% were petrol powered, 34% diesel, hybrid 4%, and pure electric 1%, while 91% of goods vehicles were diesel (Transport Analysis 2018).

The Lundby Tunnel located in Gothenburg was chosen as the test site, where the ambient particle level is elevated, compared with open city roads or highways in Gothenburg, since tunnels exhibit low dispersion and dilution of pollutants. Westbound and eastbound directions each have 2 lanes. According to the local traffic management institute, totally 26,562 vehicles passed the 2060-m-long tunnel in westbound direction in 24 h and 85% of them were personal cars driving in an average speed of 69 km/h, on April 26 in 2018 (Swedish Transport Adminstration 2018).

The measurements were performed during May 2018 until July 2018, between 08:00 and 14:00 in rain-free days, when relative humidity was lower than 70%, to maintain a suitable working environment for the measuring instruments. No major differences of the metrological parameters and no obvious traffic events or congestions were noted. Repetitions of the same test case were performed during different days with similar conditions. The test vehicle was standing inside the tunnel with engine and HVAC system on, at an uphill emergency parking spot in westbound direction, where passing vehicles can be expected to release elevated pollutants. This was to maintain long stable measurement periods for each test case, compared with driving through the tunnel which takes 2 min.

**Fig. 1:**
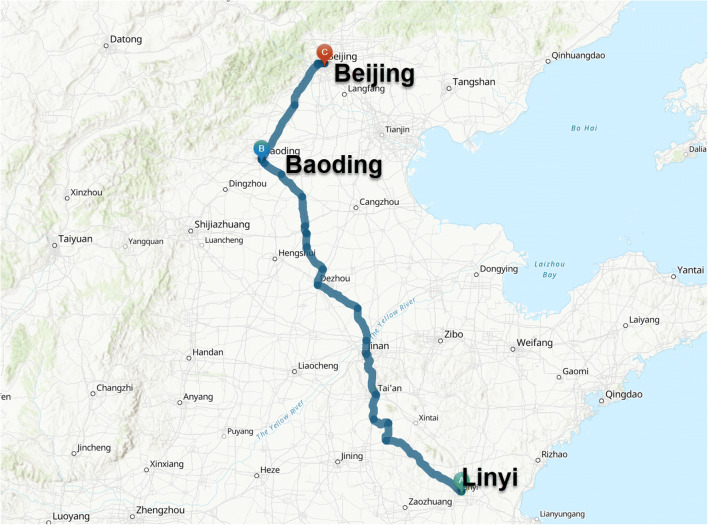
The test route in Northern China, from Linyi to Beijing, passing by Baoding

The winter measurements were performed during January 2019 driving on freeways and highways, with speeds ranging within 50–120 km/h, along the relatively polluted 760-km route from Linyi to Beijing, Northern China, as in Fig. 1. Monthly average ambient PM_2.5_ concentrations in major cities on the route at that time were, Beijing 52, Linyi 114 and Baoding 137 μg/m^3^ (CNEMC 2019). Beijing had a population of 21.54 million, and 6.08 million cars in 2018, of which 5.74 million were passenger cars (Survey Office 2018). Passenger cars accounted for around 89% of on-road vehicles in China, while goods vehicles were 11%, and 89% of all vehicles were petrol powered, 9% were diesel. Heavy goods vehicles were estimated to contribute to around 59.9% of total annual particle emission from all vehicles (MEEPRC 2018).

The measurements were carried out in two similar Volvo cars produced in 2018. The test vehicle used in Gothenburg was a PHEV (plug-in hybrid electric vehicle), Model Volvo XC90 (Table 1) with its original HVAC system. The test vehicle used in China was a diesel car, Model Volvo S90 (Table 1), with the same type of HVAC system as the PHEV used in Gothenburg.

**Table 1:** Test vehicles’ correlations and basic information

Measurement campaign	Vehicle model	Production year	HVAC system	Cabin filter	Cabin volume (m^3^)	Engine type (Power)	Mileage (km)
Gothenburg, 2018 May–July	Volvo XC90 (5-door SUV)	2018	Volvo original	New and aged synthetic filter	4.1	PHEV Petrol (2.0 L 407HP)	1706
China, 2019 January	Volvo S90 (sedan)	2018	Same as above	Same as above	2.9	Diesel (2.0 L 245HP)	5054

The filter type used in both vehicles was a multi-layer electrostatically charged synthetic filter made of polypropylene and active carbon, with dimensions of 337 × 238 × 41 mm. One newly manufactured filter and one aged filter were used in both cars. The aged filter was aged in an HVAC test rig with ducts connected to outdoor air, in 2018 April at Shanghai, where monthly average outdoor PM_2.5_ level at time was 42 μg/m^3^ (CNEMC 2018). To simulate the actual usage of filter in customer driving, ventilation fan speed was set to low (1430 rpm, around 200 kg/h), no recirculation, and ageing time was 500 h, which represents around 1-year driving, the recommended filter service interval in China.

Furthermore, both cars were equipped with an ionizer unit to study the influence of ionization on air filtration. The unit is manufactured to fit the air inlet dimensions and is installed before the water separation unit in front of the HVAC, around 50 cm upstream of the filter. It was manufactured to fit to the inlet dimensions and worked with a voltage of − 7 ± 1 kV. The high-voltage bars with sharp edges form corona discharge tips, and continuously discharge unipolar ions. Particles contained in the incoming air thus changed polarity when colliding with ions.

### Instrumentation

Particle mass and number concentration measurements were performed with Grimm MiniWRAS (Mini Wide Range Aerosol Spectrometer) model 1.371, with a log interval of 1 min. The instrument measures particles of aerodynamic diameter from 10 nm to 35 μm, distributed into 41 channels. Light scattering and electric mobility detection methods are jointly adopted, for size ranges of 0.253 to 35 μm, and 10 to 193 nm, respectively. Within a measurable mass concentration range 0.1 μg/m^3^ to 100 mg/m^3^, reproducibility of mass concentration is ± 2 μg, and ± 3% of count values (GRIMM 2019). Thus, mass and number concentration of all size channels are acquired, including PM_2.5_, UFP counts from 10 to 100 nm. Annual calibration was performed by a supplier and automatic self-test done by instrument at each startup.

**Fig. 2:**
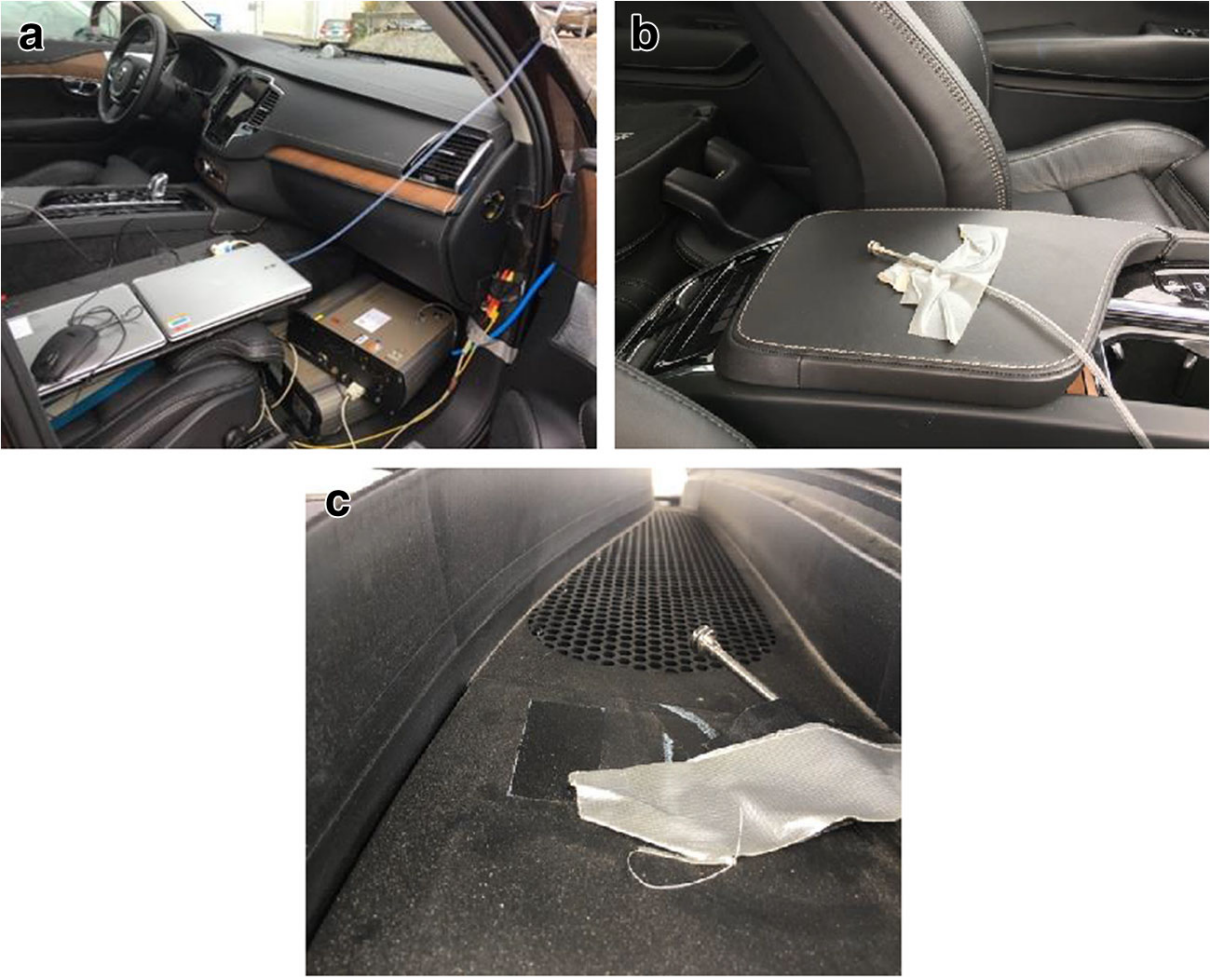
Particle instrument setup at the front row (**a**), location of the sampling tube inside the cabin (**b**), and outside sampling tube at HVAC upstream, under the wind shield, at the vehicle’s front right (**c**)

Two inter-calibrated MiniWRAS were measuring simultaneously outside and inside the cabin. The outside sampling tube was placed immediately outside of the HVAC air intake below the wind shield, which measured exactly the air at HVAC upstream. The inside sampling tube was placed above the middle armrest between the front seats, as in Fig. 2, as recommended by Abi-Esber and El-Fadel (2013). This position was chosen to measure the particle concentration in the well-mixed in-cabin air, rather than air samples at HVAC direct outlets. Two instruments started at the same time.

In addition, to investigate the possibility of ozone generation from the ionizer, 1B Technologies Model 205 Dual Beam Monitor (UV-absorption principle) was used to monitor the in-cabin ozone concentration, with a measurement frequency of 0.5 Hz and accuracy of 2% of reading above the 2.0-ppbv detection limit (1B Technologies 2019). Ozone was measured at the same place as the in-cabin particles.

Temperature, relative humidity (Rotronic Hygroflex HF534) and solar intensity sensors were mounted both inside and outside the cabin. A CO_2_ meter Vaisala Darbocap GM70 in the cabin, with the sampling head mounted on the back side of the co-pilot seat pointing to the centre of the cabin, was also used in the cabin. The testing personnel kept distance to all sampling heads throughout the measurement period, to refrain from direct breath influence.

### Measurement content and setup

To represent common driving conditions, as well as investigate PM_2.5_ concentration and UFP counts inside and outside the car under different circumstances, several climate parameters were varied and combined. Each combination of parameters, including different ventilation mode, airflow, filter, and with or without ionization, was defined as a test case, and ensured to be performed at least three times in different days. Totally, 28 test cases (explained in Table 2) equally comprise 14 baseline (no ionization) and 14 ionization cases. Considering repeated data collections, 127 of all 134 data collections were valid, which is presented in Table 3 as number of samples.

**Table 2:** Description of varied parameters in all test cases

Filter status	Ventilation airflow level ^a^	Recirculation degree (%) ^b^	Ionization
New	Xlow	0	Off^c^
Aged	Low	30	On
	Medium	50	
	High	70	

^a^Xlow (extra low) ventilation, medium ventilation and high ventilation are only used in combination with 0 recirculation

^b^Thirty percent, 50% and 70% recirculation degrees are only used in combination with low airflow

^c^Ionization off test cases means the baseline cases

**Table 3:** Summarized overall data of inside and outside PM_2.5_ concentrations, UFP counts and I/O ratio, grouped by location and ionization status

	*N* of samples	PM_2.5_ mass concentration (μg/m^3^)			UFP counts (×10^3^ particles/cm^3^)		
		In-cabin		Out.	In-cabin		Out.
		AM	AM I/O	AM	AM	AM I/O	AM
GOT baseline	40	19.8	0.36	54.9	9.24	0.37	25.8
GOT ion.	41	9.9	0.20	50.0	4.27	0.18	24.0
China baseline	23	58.1	0.35	159.7	10.8	0.40	25.0
China ion.	23	30.0	0.15	190.7	6.10	0.20	30.5

*Out.* outside, *AM* arithmetic mean, *GOT* Gothenburg, *Ion.* ionization applied cases

The baseline measurement was firstly measured as follows: the ventilation airflow rate was set to low, there was no recirculation, and both new and 500-h-aged filters were tested. Simultaneous inside and outside particle and ozone concentration levels were recorded. The climate settings during all testings were windows closed, AC on, and desired temperature of 22 °C, as well as a constant ratio of airflow at the panel and floor vents. Smoking was forbidden, and 2–3 persons sat in the vehicle. All these parameters were controlled by a software connected to the vehicle control unit, to maintain a similar environment in all measurements.

Furthermore, 3 other mechanical ventilation airflow levels (extra low (Xlow), medium, high) were also tested, to investigate the influence of the ventilation rate (estimated airflow rates at these 4 levels are around 100, 180, 260, and 380 kg/h, respectively). Three recirculation (RC) levels (30%, 50%, and 70% of total ventilation air comes from recirculation) were compared as well. Recirculation air from the vehicle cabin was mixed with incoming air and then filtered. Test cases of 4 airflow rates were always done in sequence, similar to 4 recirculation degrees, to gain similar ambient conditions when relevant cases were compared. These parameters were also controlled by the aforementioned software.

Apart from baseline testing, pre-ionization was added as another varying parameter. The ionizer was turned on, after each corresponding test case, and then the same data collection procedures were repeated. The aim was to maintain as close ambient pollutant and metrological conditions as possible for comparisons of with and without ionizer.

All the instruments were turned on at least 30 min in advance for warming up and stabilization. The ventilation parameters were varied firstly, and when a stable in-cabin air quality was achieved, a data collection interval of around 5–10 min started.

### Data analysis

Average inside and outside PM_2.5_ concentrations and UFP counts were calculated for each data collection interval firstly, by averaging the 1-min data, and then the general average was calculated for each test case (based on all data collection repetitions). The indoor to outdoor ratio (I/O ratio) of PM_2.5_ mass concentration and UFP counts were analysed afterwards, to evaluate the filtration performance regardless of ambient pollution level.

## Results

### Introduction

The introduction of the results is focused on the differences between the two measurement sites (inside a road tunnel in the City of Gothenburg, Sweden, and driving on highways between Linyi and Beijing, Northern China), and the difference between with and without pre-ionization. The results are based on PM_2.5_ concentrations and UFP counts (10–100 nm), both inside and outside the car, as well as I/O ratios, in order to facilitate comparisons with results from other studies.

To represent common driving settings in passenger cars, and increase comparability with previous particle level studies, the ventilation settings during all measurements include windows closed, AC on during the entire testing periods (22 °C), 4 levels of mechanical ventilation airflow (mainly low), and ventilation through fresh outside air or partial recirculation (mainly no recirculation).

First, average data for all test cases are summarized in Table 3, where baseline (without ionization) and ionization data are presented for Gothenburg and Northern China separately. The detailed results are found in Tables 5, 6 and 7 in the Appendix. Further on, results for different locations, baseline and with ionization, filter status (new and aged), different airflows and recirculation degrees are shown in diagrams in the following sub-chapters. The I/O ratios will only be discussed in relation to the diagrams.

Considering the measurements in Gothenburg, altogether there are 40 baseline test cases with average inside and outside PM_2.5_ concentrations of 20 and 55 μg/m^3^, and UFPs of about 9200 and 26,000 particles/cm^3^. Both inside size fractions are considerably lower applying ionization, in comparison with the baseline result, where 41 test cases present average inside and outside PM_2.5_ of 10 and 50 μg/m^3^, and UFPs of about 4300 and 24,000 particles/cm^3^. Results in China followed that trend.

Comparing results from two locations, the main difference was that outside PM_2.5_ concentrations on average were considerably higher in Northern China (baseline average 160 μg/m^3^) than in the road tunnel in Gothenburg (baseline average 55 μg/m^3^). Thereby, also the inside PM_2.5_ concentrations are on average higher in Northern China (baseline average 58 μg/m^3^) than in the road tunnel Gothenburg (baseline average 20 μg/m^3^). The relationship of I/O ratios is discussed in later diagrams. Besides, the average outside temperature during measurements was 24.8 and 3.3 °C in Gothenburg and China respectively, and corresponding relative humidity was 54% and 26%. The solar intensity in China was 257 W/m^2^, and that in Gothenburg was around zero due to the tunnel environment.

### Location and filter status

The most important results presented in the introduction are here presented in more detail using diagrams showing PM_2.5_ concentrations, UFP counts and the corresponding I/O ratios.

**Fig. 3:**
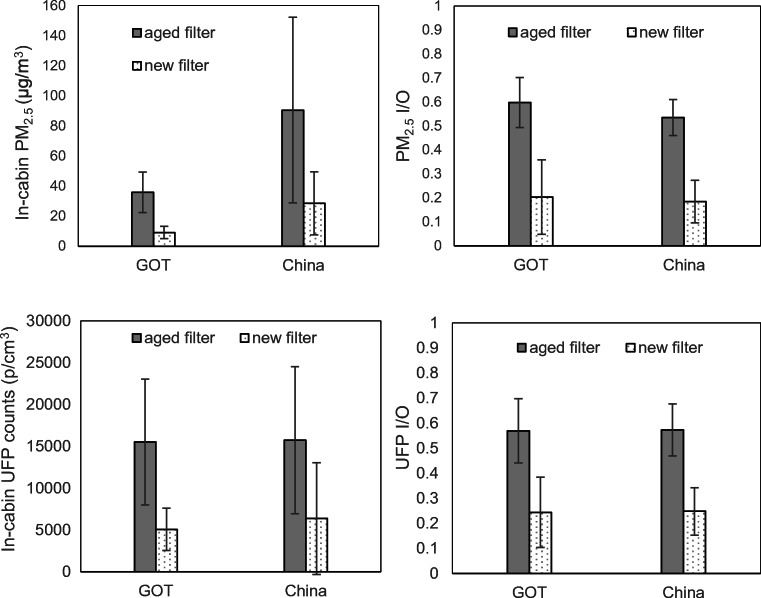
Comparison of all baseline (no ionization) cases in-cabin PM_2.5_ concentration, in-cabin UFP counts and I/O ratios, grouped by locations, new and aged filter. Error bars present standard deviation. GOT: Gothenburg

Figure 3 shows comparisons between inside PM_2.5_ concentrations and inside UFP counts for the two locations, together with corresponding I/O ratios. The cases included are all the baseline cases, i.e. no ionization utilized. The already mentioned differences between inside PM_2.5_ concentrations for the two locations are clearly shown. The UFP counts are however of the same order in both locations. An independent-sample *t* test shows no statistically significant difference of inside UFP counts, neither for new nor for aged filters (*p* = 0.52 and 0.94, respectively).

The differences of using new and aged filters are also clearly shown. The inside PM_2.5_ concentration and the UFP counts are about three times higher using an aged filter in comparison with a new filter.

I/O ratios are further compared, to gain understanding of the system filtration performance. We can notice that the I/O ratios are almost equal across two locations, when the same filter is used, compared with the difference found in PM_2.5_ concentrations and UFP counts. One main reason is that the same filters and HVAC system are used for both locations, and the main protection against particles comes from the HVAC, or specifically the filter. The overall baseline case average of 0.36 in Gothenburg and 0.35 in China (see Table 3) shows a relatively effective HVAC system regardless of ambient condition. While when new and aged filters were compared, in both locations around a triple I/O ratio is presented for the aged filter. The reduction of performance was apparent when the aged filter was mounted.

**Fig. 4:**
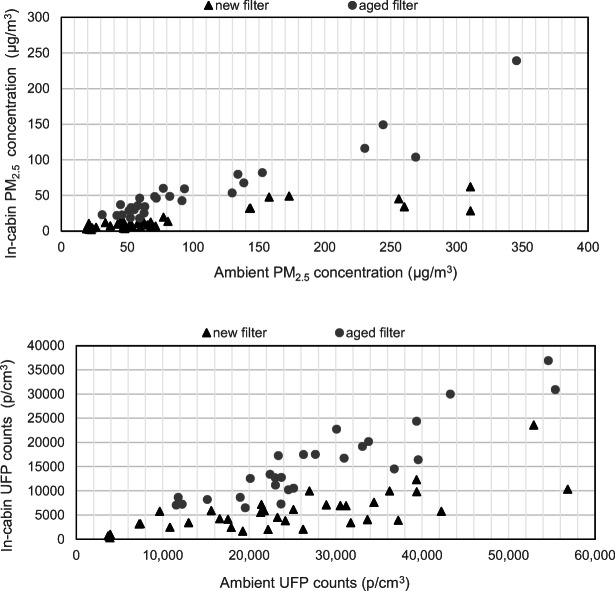
Correlations between average in-cabin and outside PM_2.5_ concentration and UFP counts, grouped by new and aged filters. Samples include all baseline (no ionization) cases

Further, we compare all 63 individual trip averages from the baseline testing (the baseline samples in Table 3). The Pearson correlation coefficient (*r*) is calculated between in-cabin and outside PM_2.5_ or UFP using software SPSS, for each filter type. All groups show statistical significance (all *p* value < 0.01). The correlations between in-cabin and ambient PM_2.5_ values are strong (*r* = 0.866, 0.945 for new and aged filters) as shown in Fig. 4. Clearly, the aged filter resulted in higher in-cabin PM_2.5_ and UFP counts. When dust was loaded on the surface and within the filter material and the electrostatic characteristics reduced, the filtration capabilities are diminished. Similarly, Huang et al. (2012) stated that high correlations (*r* = 0.863) were observed between PM_2.5_ concentrations while commuting and PM_2.5_ concentrations at the fixed monitoring site. Jain (2017) reported a significant correlation between ambient and in-vehicle concentrations for PM_2.5_.

The UFP counts followed similar trends with slightly lower correlations (*r* = 0.735, 0.904 for new and aged filters). Likewise, Zhu et al. (2007) mentioned that in-cabin UFPs follow outdoor with a 30-s delay, but do not change as sharp as outside UFPs, while inside UFPs still follow the trend of outside UFPs when comparing different highways.

### Utilization of pre-ionization

The most important results for PM_2.5_ concentrations and inside UFP counts with ionization applied presented in the introduction are here presented in more detail using diagrams showing the corresponding I/O ratios.

**Fig. 5:**
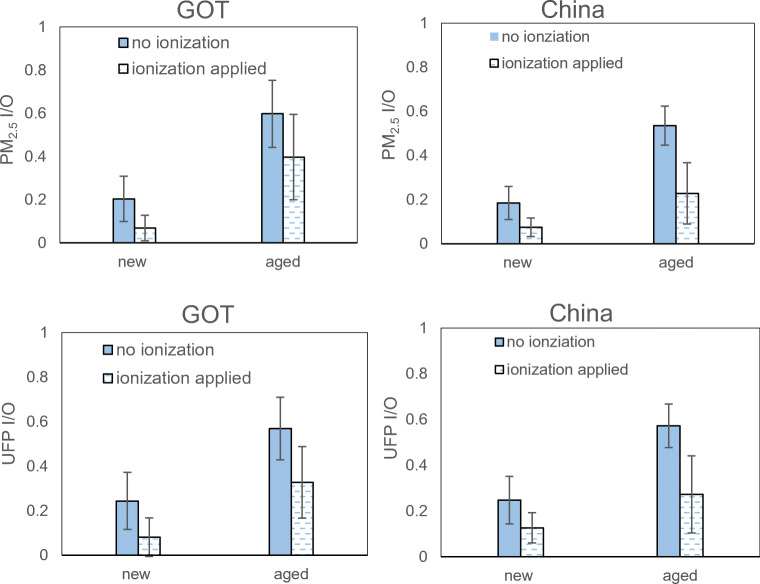
Influence of pre-ionization on in-cabin PM_2.5_, UFP, and I/O ratio in Gothenburg and China, grouped by new and aged filters. Samples include all test cases. Error bars present standard deviation. GOT: Gothenburg

Figure 5 shows comparisons of I/O ratios, between all baseline tests and all ionization tests. All the ionization tests were performed directly after corresponding the baseline test of the same ventilation settings. Thus, the comparison groups share the same distribution in different airflow and recirculation levels, expect for one sample which was invalid due to instrument error. The main result is that there is a considerable reduction of the I/O ratios for both PM_2.5_ and UFP and for both new and used filters, in both locations.

The new filter demonstrates more stable performance in the two locations, with and without pre-ionization, and the PM_2.5_ I/O ratio decreased by 0.13 and 0.11 respectively. The enhancement for the aged filter is more distinct and observed to relate with locations. The PM_2.5_ I/O ratio decreased by 0.20 and 0.31 in Gothenburg and China correspondingly, although with higher fluctuations. More importantly, ionization applied to a used filter makes it almost comparable with a new filter without ionization. Four comparisons were made between the I/O ratios of the aged filter with ionization and the new filter without ionization, for two locations and two particle sizes respectively. The comparisons show no significant difference in I/O ratios (*p* > 0.05), only except for the comparison of the PM_2.5_ I/O ratio in Gothenburg which shows a significant difference (*p* < 0.05).

Regardless of ionization, the trend of slightly higher I/O ratio in Gothenburg was maintained for both filters, which was probably partially attributed to extremely high ambient concentrations in most test days in Northern China**.**

It should however be noted that the standard deviation between the different test cases is larger for aged than for new filters and this could possibly be related to that the aged filter has uneven dust loading. There is a considerable overlap between PM_2.5_ and UFP I/O ratios in Gothenburg compared with China, and particle size distribution would possibly be an important influencer.

The measurements of inside and outside ozone levels show that the influence of ionization on in-cabin ozone concentration was negligible, supporting that Kim et al. (2017b) considered that an ionizer with sharp geometry tips and multiple electrodes generates low ozone emission. The average outside ozone concentration was 9.9 ppb in Gothenburg, and that of the inside was 2.2 ppb. This low value is because of less sunlight in the tunnel. Corresponding values in China were 42.8 and 8.4 ppb.

### Ventilation airflow and recirculation

The results for different airflows and different degrees of recirculation are here presented in more detail in diagrams showing I/O ratios. The cases included are shown in Table 5 in the Appendix, in which the subgroup of airflow and recirculation degree describes how these two parameters distribute in samples. Only results from baseline (no ionization) and ionization measurements in Gothenburg are presented as they are more comprehensive, and that the same tendencies are found in the measurements in Northern China and in several references (Zhu et al. 2007; Xu and Zhu 2009; Knibbs et al. 2010).

**Fig. 6:**
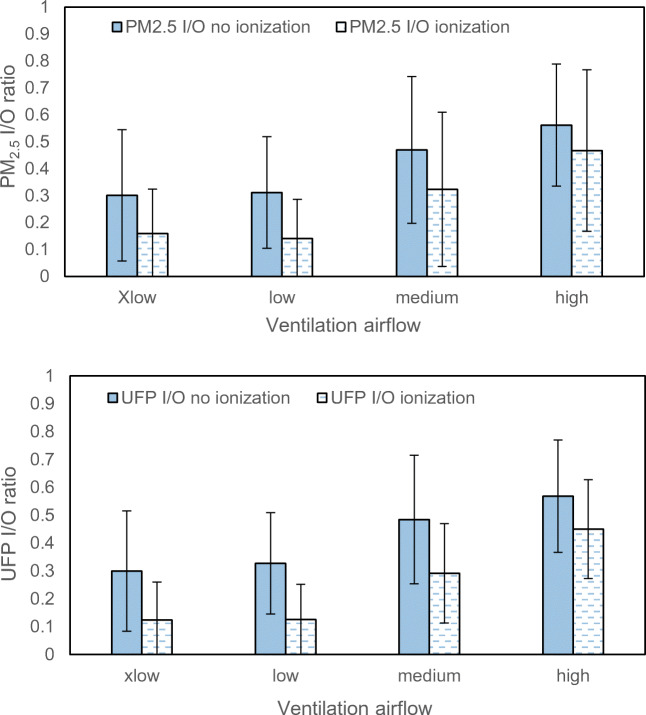
Influence of 4 ventilation airflow levels on the I/O ratio of PM_2.5_ and UFP counts. Samples include all Gothenburg measurements (baseline and ionization). Error bars present standard deviation

**Fig. 7:**
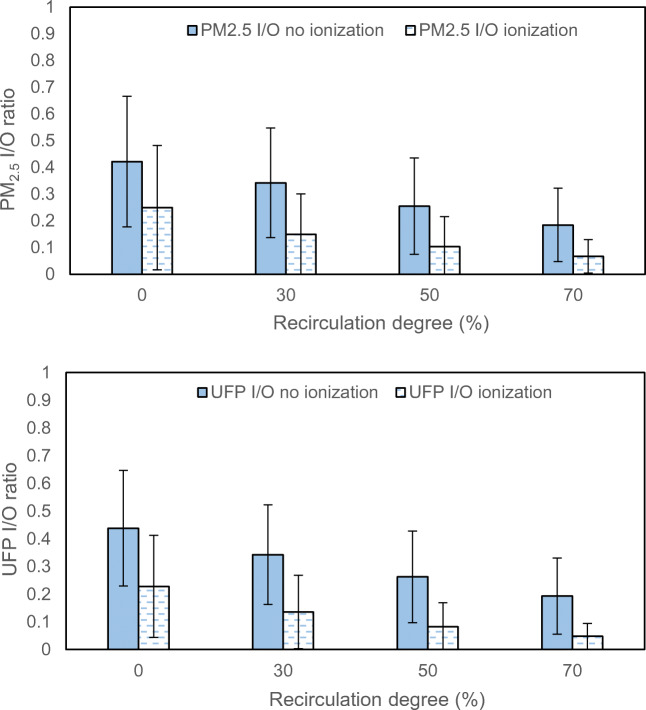
Influence of 4 recirculation degrees on the I/O ratio of PM_2.5_ and UFP counts. Samples include all Gothenburg measurements (baseline and ionization). Error bars present standard deviation

Figure 6 shows the measurement results for four mechanical ventilation airflow rates, extra low, low, medium and high, where low airflow is used in the majority of test cases. Figure 7 shows the measurement results for four recirculation degrees, 0, 30%, 50% and 70%, where no (0) recirculation is used in the majority of test cases.

The measurements show the expected tendencies, i.e. increased airflow will result in increased I/O ratio and increased recirculation degree reduced the I/O ratio. The relatively large variation possibly stemmed from lack of sufficient sample points, and more importantly, new and aged filter samples are combined.

Low and medium airflow settings are prevailing in vehicles during normal customer driving. When the fan setting was high, a doubled PM_2.5_ I/O ratio appeared in both particle fractions, and the elevation was much more obvious compared with Xlow and low level, since shorter residence time deteriorated particle collection capability through interception, inertial impaction, diffusion and electrostatic attraction (Qi et al. 2008).

When ionization is applied, the varying airflow also showed influence, that the reduction of I/O ratio from ionization was less significant at higher airflow due to less contact time of particles with ions prior to filtration (Park et al. 2011).

Seventy percent recirculation resulted in I/O and UFP counts which became less than half compared with no recirculation. The in-cabin PM_2.5_ decreased to below 10 μg/m^3^. One important reason is that recirculated air from the cabin contains less particles than outside air, and is further mixed with outside air, then filtered again.

## Discussion

The averages presented in this study are based on several parameter variations. The main advantage is that they represent the result under normal variations in driving conditions. However, the averages presented for China are e.g. influenced to a larger extent by different airflows and different recirculation degrees than the averages presented from Gothenburg, due to few test cases for the normal low airflow.

It is acknowledged that the results are only conclusive related to the influence of filter status (new and aged) and a new ionization unit, based on the measurement procedure applied. The results in general show rather high standard deviations, due to the outdoor conditions and several ventilation parameters varying between data collections.

The study shows that the filter status (new or aged) has a major influence on the ability of the HVAC system to capture particles. However, these results are valid for a specific type of filter brand used by a specific car brand and aged in a specific way. Different dust loading status, filter design and material can be expected to augment the variance. Although it is likely that the same results will appear for other filter types under similar conditions, it will be difficult to compare the results due to the lack of common vehicle filter standards. This might also be the reason that the filter type and status are only mentioned briefly in some of the references.

Furthermore, the influence of particle size distributions on the results has not been evaluated, to not include too much in the same article and take the focus from the main results related to filter status and ionization. For example, the outside PM_2.5_ concentration in two locations is obviously different, but outside UFP counts are quite close. Further analysis of particle size distribution from measurement results is thus of interest, not only to understand particle correlations but also to understand the influence of local transport conditions.

The baseline measurement results from this study are listed together with the main results presented in 10 references for a comparison in Table 8 in the Appendix. The two main differences between our study and these studies are (1) the filter status (new and aged) and ionization and (2) parallel measurements of inside and outside PM_2.5_ concentrations and UFP counts, for all test cases. Most of the publications do not contain information regarding filter status. However, a few of them state that the tested vehicle had filters without replacement since purchase, i.e. highly aged filter.

The in-cabin particle PM_2.5_ concentrations are reported in 7 out of 10 references, while only 2 rather specific studies reported PM_2.5_ I/O and/or outside PM_2.5_ concentrations. The results for in-cabin particle PM_2.5_ concentrations presented in this study generally indicate lower or similar concentrations and higher performances (lower I/O ratios); especially, our results are closer to studies on newer vehicles.

The in-cabin UFP counts are reported in 6 out of 10 references, while only 3 rather specific studies reported UFP counts I/O and/or outside UFP counts. The results for in-cabin UFP counts presented in this study generally indicate lower counts and similar or higher performances (I/O ratios). The outside UFP counts are however much higher in some references.

Our findings also presented effectiveness of ionization-assisted filtration in vehicle context. When comparing the improvement on the particle filtration, the results from car measurements are relatively less prominent than single filter tests (Agranovski et al. 2006; Park et al. 2011; Shi 2012). Likely reasons are that system efficiency and component efficiency are compared, and the car cabin itself combined with the connected HVAC system is a less enclosed environment then test rigs or chambers. Air infiltration and leakage could contribute. In addition, the vehicle’s entire system also includes the HVAC ducts, material surfaces and ground mats, which constitute potential particle deposition locations.

## Conclusion

Measurements of in-cabin and outside PM_2.5_ concentration and UFP counts were performed, firstly in a road tunnel in Gothenburg, Sweden, and afterwards on roads in Northern China, in order to study the influence of different outside conditions on the performance of the studied HVAC system. The conclusion is here that the HVAC system performance is very similar for the two outside conditions, with average I/O ratios of the order of 0.35–0.40 for baseline conditions and of the order of 0.15–0.20 with ionization applied.

The main aim was then to evaluate the influence of filter status (new and aged), without and with pre-ionization, using the same HVAC system.

First, the aged filter clearly worsened the particle filtration ability, between two to three times, for both in-cabin PM_2.5_ and UFP counts, in both test locations, compared with the new filter of the same type. As an example, the average PM_2.5_ concentration I/O ratio for all baseline variations in Gothenburg is 0.36, while the average for the aged filter is 0.60 and the average for the new filter is 0.20. Moreover, the corresponding UFP I/O ratio values were 0.57, 0.24.

Second, ionization upstream of the cabin filter clearly improved the particle filtration ability, for both in-cabin PM_2.5_ and UFP counts, in both locations, compared with no ionization upstream of the filter. On average, the I/O was decreased by around 0.15–0.20. The results also indicate that ionization combined with an aged filter could result in a performance similar like that of a new filter without ionization applied.

Third, the test cases with varying ventilation air flows and varying recirculation degrees show expected results. The I/O ratios increase with increased air flow and decrease with increased recirculation degree.

## Appendix

**Table 4:** Summary of comparative PM_2.5_ and UFP studies in vehicles

Study	Test location and routes	Vehicle type and filter status	Ventilation setting	Measured pollutant	Instrument
(Kaur et al. 2005)	London, UK; (1) Heavier route (2) A circuit with little traffic in central London	Toyota Starlet 1996, petrol.	Ventilation as normal	PM_2.5_ and UFP counts (0.02–1 μm) in cabin	Gravimetric sampler, and TSI P-TRAK model 8525
(Zhu et al. 2007)	Los Angeles, USA; 4 different LA highways	Three private cars, all with manufacturer-equipped filter with active carbon	Windows all closed, AC on when fan is on (1) Fan off and RC off; (2) Fan on and RC off; (3) Fan on and RC on.	UFP counts in cabin	Two TSI model 3785 WCPC, and two TSI model 3080 scanning mobility particle sizers
(Knibbs et al. 2009)	Sydney, Australia; a twin bore tunnel	Unleaded private vehicles	/	UFP outside (in the tunnel)	TSI 3007 CPC
(Knibbs et al. 2010)	Sydney, Australia; a twin bore tunnel	5 representative passenger vehicles, 2 cars have original pollen filter (RC air not filtered), the rest have no filter	(1) RC off, fan on lowest (2) RC off, fan at 2nd-highest (3) RC on, fan at lowest (4) RC on, fan off, (123) AC on full cooling (4) AC off	UFP counts in cabin	One TSI 3007 CPC
(Knibbs and de Dear 2010)	Sydney, Australia; a twin bore tunnel	Mitsubishi Magna 1998, petrol, no filter	AC on cool, lowest fan, no RC	PM_2.5_ and UFP counts in cabin	TSI 8520 (overestimate) and TSI 3007
(Huang et al. 2012)	Beijing, China; 2 routes sharing same start and end points: (1) Broader, higher traffic flow, less intersections; (2) Narrower, heavy congestions	Gasoline taxi	AC on, windows closed	PM_2.5_ in cabin	Beijing Green Tech. Spectrometer model LD-6S
(Abi-Esber and El-Fadel 2013)	Beirut, Lebanon; (1) A circuit in commercial/residential area (2) Highway	6 different passenger cars, original filter never replaced since purchase	(1) W1/2O (2) AC on RC (3) AC on OSA	PM_2.5_ in and out of cabin	TSI DustTrak
(Both et al. 2013)	Jakarta, Indonesia	/	AC on/off	PM_2.5_ and UFP counts in cabin	TSI DustTrak 8520, light scattering photometer and P-TRAK model 8525
(Qiu et al. 2017)	Xi’an, China; One major city road	Gasoline car	(1) WC fan off (2) WC AC on RC (3) WC AC on OSA (4) W1/2O, fan off	PM_2.5_ in cabin	Grimm aerosol monitor 1.109
(Jain 2017)	Delhi, India a ~ 15-km long ring road	/	AC and non-AC cars	PM_2.5_ in cabin	Grimm aerosol monitor 1.108
This study	Gothenburg, Sweden, city tunnel and Northern China highway.	Volvo PHEV XC90 and Diesel V60 2018, New and pre-aged filters, the same filter type as the vehicle original	(1) AC on OSA (4 airflow levels), RC off (2) AC on OSA, RC on (4 RC levels)	PM_2.5_ and UFP counts in and out of cabin	Grimm MiniWRAS Wide Range Aerosol Spectrometer model 1.371

*AC* air conditioning, *CPC/WCPC* (water-based) condensation particle counter, *OSA* outside air, *RC* recirculation, *UFP* ultrafine particle, *WC* window closed, *W1/2O* window half opened

**Table 5:** Gothenburg baseline (no ionization) data. Summarized data of inside and outside PM_2.5_ concentrations, UFP counts and I/O ratio, grouped by filter status, ventilation airflow and recirculation degree

	*N*	PM_2.5_ mass concentration (μg/m^3^)					UFP counts (particles/cm^3^)				
		In-cabin				Out.	In-cabin				Out.
		AM	Range (min–max)	AM I/O	Range (min–max)	AM	AM	Range (min–max)	AM I/O	Range (min–max)	AM
All samples	40	19.8	3.6–60.1	0.36	0.07–0.83	54.9	9241	1687–30,913	0.37	0.08–0.76	25,816
Filter status											
New	24	9.2	3.6–19.4	0.20	0.07–0.50	50.6	5056	1687–10,332	0.24	0.08–0.61	24,401
Aged	16	35.8	18.0–60.1	0.60	0.31–0.83	61.2	15,519	6497–30,913	0.57	0.33–0.76	27,939
Ventilation airflow											
Xlow	5	19.1	4.5–42.5	0.30	0.10–0.64	62.6	7931	2469–16,408	0.30	0.11–0.61	28,363
Low	25	18.5	3.6–59.2	0.31	0.07–0.70	57.7	8715	1687–30,913	0.33	0.08–0.67	27,405
Medium	5	22.8	6.8–60.1	0.47	0.19–0.78	46.7	10,652	3196–30,006	0.48	0.26–0.76	21,111
High	5	24.3	10.6–46.0	0.56	0.35–0.83	41.0	11,773	5746–22,761	0.57	0.34–0.76	20,030
Recirculation degree (%)											
0	25	23.4	4.5–60.1	0.42	0.10–0.83	54.6	10,987	2469–30,913	0.44	0.11–0.76	25,843
30	5	18.4	9.8–30.0	0.34	0.17–0.57	57.7	9179	4488–12,792	0.34	0.19–0.57	31,159
50	5	13.7	4.8–25.1	0.25	0.11–0.49	55.0	5899	2432–10,547	0.26	0.12–0.46	23,976
70	5	9.8	3.6–18.5	0.18	0.07–0.36	53.5	3914	1687–7280	0.19	0.08–0.35	22,176

*AM* arithmetic mean, *I/O* indoor to outdoor ratio, *N* number of samples, *Out.* outside, *UFP* ultrafine particle, *Xlow* extra low

**Table 6:** Gothenburg ionization applied data. Summarized data of inside and outside PM_2.5_ concentrations, UFP counts and I/O ratio, grouped by filter status, ventilation airflow and recirculation degree

	PM_2.5_ mass concentration(μg/m^3^)						UFP counts (particles/cm^3^)				
	In-cabin					Out.	In-cabin				Out.
	*N*	AM	Range (min–max)	AM I/O	Range (min–max)	AM	AM	Range (min–max)	AM I/O	Range (min–max)	AM
All samples	41	9.9	0.8–46.0	0.20	0.01–0.74	50.0	4274	157–19,220	0.18	0.01–0.61	24,038
Filter status											
New	25	2.8	0.8–8.2	0.07	0.01–0.23	50.6	1420	157–5975	0.08	0.01–0.33	22,403
Aged	16	21.0	4.8–46.0	0.40	0.12–0.74	61.2	8732	1650–19,220	0.33	0.09–0.61	26,593
Ventilation airflow											
Xlow	5	7.7	1.1–19.0	0.16	0.02–0.40	62.6	3076	397–8365	0.12	0.02–0.34	25,390
Low	27	7.0	0.8–30.5	0.14	0.01–0.47	57.7	2780	157–12,368	0.13	0.01–0.42	23,835
Medium	5	17.2	2.1–43.4	0.32	0.08–0.73	46.7	8322	1130–19,220	0.29	0.09–0.52	25,293
High	4	23.3	3.4–46.0	0.47	0.18–0.74	41.0	10,793	1920–18,945	0.45	0.26–0.61	22,148
Recirculation degree (%)											
0	26	12.5	1.1–46.0	0.25	0.02–0.74	54.6	5596	397–19,220	0.23	0.02–0.61	23,450
30	5	7.8	1.3–20.1	0.15	0.02–0.34	57.7	2990	524–7222	0.14	0.02–0.30	24,352
50	5	5.3	0.8–14.2	0.10	0.01–0.26	55.0	1866	321–5048	0.08	0.01–0.18	27,868
70	5	3.1	0.8–7.4	0.07	0.02–0.15	53.5	1090	157–2842	0.05	0.01–0.10	22,953

*AM* arithmetic mean, *I/O* indoor to outdoor ratio, *N* number of samples, *Out.* outside, *UFP* ultrafine particle, *Xlow* extra low

**Table 7:** China baseline and ionization data. Summarized data of inside and outside PM_2.5_ concentration, UFP counts and I/O ratio, grouped by filter status

	PM_2.5_ mass concentration(μg/m^3^)						UFP counts (particles/cm^3^)				
	In-cabin					Out.	In-cabin				Out.
	*N*	AM	Range (min–max)	AM I/O	Range (min–max)	AM	AM	Range (min–max)	AM I/O	Range(min–max)	AM
Baseline											
All baseline samples	23	58.1	1.8–239.0	0.35	0.08–0.69	159.7	10,843	312–36,885	0.40	0.08–0.68	24,966
Filter status											
New	12	28.5	1.8–62.0	0.18	0.08–0.31	153.3	6362	312–23,615	0.25	0.08–0.44	22,672
Aged	11	90.5	21.8–239.0	0.54	0.39–0.69	166.6	15,731	7067–36,885	0.57	0.40–0.68	27,469
Ionization applied											
All ionization samples	23	30.0	3.5–127.2	0.15	0.02–0.46	190.7	6101	957–31,279	0.20	0.05–0.55	30,534
Filter status											
New	11	14.5	5.1–30.2	0.07	0.02–0.15	200.1	3019	957–5163	0.13	0.05–0.25	25,752
Aged	12	44.3	3.5–127.2	0.23	0.05–0.46	182.1	8926	1319–31,279	0.27	0.05–0.55	34,917

*AM* arithmetic mean, *I/O* indoor to outdoor ratio, *N* number of samples, *Out.* outside, *UFP* ultrafine particle

**Table 8:** Summarized measurement results of comparative studies on PM_2.5_ and UFP (including this study). Detailed information of these studies is listed in Table 4

**PM**_**2.5**_					
Reference	**Relevant ventilation conditions**^**a**^	**PM**_**2.5**_**in cabin (μg/m**^**3**^**)**		**PM**_**2.5**_**I/O**	**PM**_**2.5**_**outside (μg/m**^**3**^**)**
(Kaur et al. 2005)	Private car, ventilation as normal	AM = 38.07 ±14.1 (sample *N* = 29)		/	/
(Knibbs and de Dear 2010)	AC on and cool the cabin, lowest fan speed, no RC	GM: 22.6, AM: 27.3		/	/
(Huang et al. 2012)	taxi's AC on, window closed	31.64 ± 20.77		Median: 0.79 to 1.04	FMS: 35.15 ± 23.97
(Abi-Esber and El-Fadel 2013) (*results presented for mobile tests)*	window closed, AC on OSA, fan medium	79 ± 34		Above 1 (~1.1)	/
	window closed, AC on RC, fan medium	38 ± 28		Below 1 (~0.5)	/
(Both et al. 2013)	private car AC and non-AC	mean (SD) = 91 (38)		/	/
(Qiu et al. 2017)	window closed, AC on RC	Morning: Afternoon:	10.09 ± 6.63 8.95 ± 1.08	/	/
	window closed, AC on OSA	Morning: Afternoon:	22.06 ± 3.61 14.56 ± 10.86	/	/
(Jain 2017)	AC private car	roughly around 80		/	/
This study GOT baseline^b^	Fan on 2^nd^ level, RC off new filter	AM 9.2		AM 0.20	AM 50.6
	Fan on 2^nd^ level, RC off aged filter	AM 35.8		AM 0.60	AM 61.2
This study China baseline^b^	Fan on 2^nd^ level, RC off new filter	AM 28.5		AM 0.18	AM 153.3
	Fan on 2^nd^ level, RC off aged filter	AM 90.5		AM 0.54	AM 166.6
**UFP**					
**Reference**	**Relevant ventilation conditions**^**a**^	**UFP in cabin (× 10**^**3**^**p/cm**^**3**^**)**		**UFP I/O ratio**	**UFP outside (×10**^**3**^**p/cm**^**3**^**)**
(Kaur et al. 2005)	Private car, ventilation as normal	AM=99.7/cm^3^ range 36.5-151.81 (sample *N* = 13)		/	/
(Zhu et al. 2007) (*results presented for different locations, newest vehicle model)*	Fan off RC off	9.1± 33 to 150 ±34		~0.15	22 ± 24 to 256 ± 119
	Fan on RC off			~0.3	
	Fan on RC on.			~0.05	
(Knibbs et al. 2009)	Private car driving in a tunnel	/		/	Average:6000 Median:1700
	Private car driving in mixed city routes	/		/	Median: 160
(Knibbs et al. 2010) *(Results presented for 2 vehicles fitted with HVAC and filter, separated with)*	RC off, fan lowest, AC full cooling	Mean 282;127		Median 0.66;0.88	/
	RC off, fan 2^nd^-highest, AC full cooling	Mean 611;257		Median 0.84;0.91	/
	RC on, fan lowest, AC full cooling	Mean 34;40		Median 0.08;0.45	/
(Knibbs and de Dear 2010)	AC on cooling, fan lowest, no RC	GM 75 AM 89		/	/
(Both et al. 2013)	private car AC and non-AC	Median Non-AC:~400 AC:~140		/	/
This study GOT baseline ^b,c^	Fan 2^nd^ low, RC off new filter	AM 5.1		AM 0.24	AM 24.4
	Fan 2^nd^ low, RC off aged filter	AM 15.5		AM 0.57	AM 27.9
This study China baseline ^b,c^	Fan 2^nd^ low, RC off new filter	AM 6.4		AM 0.25	AM 22.6
	Fan 2^nd^ low, RC off aged filter	AM 15.7		AM 0.57	AM 27.5

*AM* arithmetic mean, *AC* air conditioning, *FMS* fixed monitoring site, *GOT* Gothenburg, *GM* gravimetric mean, *N* number of samples, *OSA* outside air, *RC* recirculation, *UFP* ultrafine particle, *SD* standard deviation

^a:^Relevant ventilation conditions to this study are selected from corresponding studies summarized in Table 4

^b^Baseline measurements mean test cases without ionization

^c^UFP size range 10–100 nm
